# Leaky expression of channelrhodopsin-2 (ChR2) in Ai32 mouse lines

**DOI:** 10.1371/journal.pone.0213326

**Published:** 2019-03-26

**Authors:** Arthi Prabhakar, Dragan Vujovic, Lian Cui, William Olson, Wenqin Luo

**Affiliations:** 1 University of Texas at Dallas, Dallas, TX, United States of America; 2 Williams College, Williamstown, MA, United States of America; 3 Department of Neuroscience, University of Pennsylvania, Philadelphia, PA, United States of America; Baylor College of Medicine, UNITED STATES

## Abstract

Optogenetics enables the selective activation of genetically-targeted neuronal populations using light-sensitive ion channels. Genetic strategies using Cre-dependent mouse strains, especially the Ai32 line expressing Channelrhodopsin (ChR2)-EYFP fusion protein, have been a popular means to drive opsin expression in a cell-type specific manner. Here we report a low level of leaky ‘off-target’ (Cre-independent) ChR2-EYFP expression in Ai32/Ai32 homozygous mice throughout the nervous system. This leaky off-target expression was characterized in multiple prevalent nervous system regions using anti-EYFP immunostaining. Expression of full-length ChR2-EYFP protein was confirmed using immunoprecipitation followed by Western blotting. Notably, light stimulation of these ChR2-EYFP expressing neurons in the spinal cord dorsal horn did not induce detectable photocurrents in juvenile 4-week old mice. Given the wide use of the Ai32 line by many labs, our results suggest researchers should be vigilant of possible off-target ChR2-EYFP expression in their region of interest, especially when generating Ai32/Ai32 homozygotes to drive high levels of ChR2-EYFP expression in adult mice.

## Introduction

Optogenetics has revolutionized neuroscience by allowing for selective activation of genetically-targeted neuronal populations using light. In this technique, target cells are driven to express light-sensitive opsins, which are commonly ion channels. For example, Channelrhodopsin-2 (ChR2) is a blue light-gated non-specific cation channel that drives neuronal activation [1]. Blue light illumination in ChR2 expressing animals allows for specific, temporally precise control of neuronal activity in a wide range of *in vitro* and *in vivo* contexts [2].

Genetic strategies using Cre-dependent mouse strains have been a popular means to drive opsin expression in a cell-type specific manner. For example, using a *Rosa* knock-in loxP-STOP-loxP strategy that allows for high-level, specific transgene expression [3], Madisen et al engineered multiple mouse lines for Cre-dependent, robust expression of opsins [4]. These lines are very useful reagents for the neuroscience research community. One of these lines, the Ai32 line expressing a ChR2-EYFP fusion protein, has been widely used for cell-type specific expression of ChR2. Following the cassette design for the *Rosa* knock-in allele, the Ai32 uses the CAG promoter and a woodchuck hepatitis virus posttranscriptional regulatory element (WPRE) to drive high levels of ChR2-EYFP expression [3,4]. In the original paper, the authors reported that, while other lines showed some background (Cre-independent) expression of opsin mRNA, the Ai32 line does not express ChR2-EYFP in the absence of Cre recombinase.

In our research, we occasionally noticed ChR2-EYFP expression in Cre-positive Ai32 mice in a manner that was not predicated based on Cre-line recombination pattern. We therefore sought to examine the possibility of off-target, or “leaky”, expression of ChR2-EYFP in Ai32/Ai32 homozygous mice in the absence of Cre. We performed immunostaining for EYFP of Ai32/Ai32 mice and indeed found leaky ChR2-EYFP expression throughout the nervous system. We also confirmed that this EYFP signal corresponds to full length ChR2-EYFP protein using immunoprecipitation. Our results suggest that the strong gene expression driven by this *Rosa* cassette can result in background off-target expression of ChR2-EYFP, especially in Ai32/Ai32 homozygous mice. Nevertheless, light stimulation of these ChR2-EYFP expressing neurons in dorsal horn of the spinal cord slices did not induce detectable photocurrents in 4-week old juvenile mice. In short, our study clearly showed background expression of ChR2-EYFP in Ai32 homozygous mice. Given the popularity of the Ai32 line, this finding suggests that researchers using this line should be vigilant of possible off-target ChR2-EYFP expression in their region of interest, especially in adult mice as ChR2-EFYP expression level accumulates with age.

## Materials and methods

### Mouse strains

Mice were raised in a barrier facility in Hill Pavilion, the University of Pennsylvania. All procedures were conducted according to animal protocols approved by Institutional Animal Care and Use Committee (IACUC) (Protocol:804886) of the University of Pennsylvania and National Institutes of Health guidelines. Mice used in this paper were initially purchased from Jackson Labs or Charles River Laboratories and were subsequently propagated by our lab, and have been described previously: Ai32 *Rosa*^*ChR2(H134R)-EYFP*^ (IMSR Cat# JAX:024109, RRID:IMSR_JAX:024109), *OMP*^*Cre*^ (IMSR Cat# JAX:006668, RRID:IMSR_JAX:006668), CD1 (IMSR Cat# CRL:22, RRID:IMSR_CRL:22).

### Immunostaining

Procedures were conducted as previously described [5]. Briefly, mice (>6 weeks old) used for immunostaining were anesthetized with ketamine/xylazine/acepromazine and transcardially perfused with 4% PFA/PBS, and dissected tissue (brain, spinal cord, or DRGs) was post-fixed for 2 hr-overnight in 4% PFA/PBS at 4° C. Tissue used for immunostaining was then sectioned (100 μm) using a T1200S vibratome (Leica Microsystems, Nussloch, Germany). Immunostaining of sections was performed as described previously [5,6]. The following primary antibodies were used: chicken anti-GFAP (Aves Labs Cat# GFAP, RRID:AB_2313547), chicken anti-GFP (Aves Labs Cat# GFP-1020, RRID:AB_10000240), rabbit anti-GFP (Thermo Fisher Scientific Cat# A-11122, RRID:AB_221569), mouse anti-NeuN (Millipore Cat# MAB377, RRID:AB_2298772).

### Biochemistry

Olfactory bulbs (~20–30 mg) of CO_2_ euthanized adult (3–6 month old) *Omp*^*Cre*^*;Ai32/Ai32*, *Ai32/Ai32*, or WT control mice were dissected out and snap frozen on dry ice. Samples were lysed with 1.4 mL of ice cold RIPA buffer (50 mM Tris pH = 8, 150 mM NaCl, 1% NP-40, 0.5% sodium deoxychoate, 0.1% SDS) with added protease inhibitors (Sigma, P8340, St. Louis, MO) and were homogenized with a handheld rotor fixed with a sterile pestle. Sample were rocked for 2 hours at 4 degrees and centrifuged for 20 min at 12,000 rpm at 4 degrees. Supernatant was collected and moved to a fresh tube.

Following tissue lysis, ChR2-EYFP protein was concentrated through immunoprecipitation. 1 mL of cell lysate was incubated with 2 uL (1:500) rabbit anti-GFP (Molecular Probes Cat# A-11122, RRID:AB_221569, Carlsbad, CA). Following this, 10 uL of Protein G agarose conjugate suspension (EMD MilliporeSigma, 16–201, St. Louis, MO) was added, and tubes were rocked at 4° C overnight. Beads were then collected by centrifugation at 1,000xg for 30 seconds at 4° C, and supernatant was discarded. Beads were washed 3 times with RIPA buffer with protease inhibitors Beads were then resuspended in 40 μl of 2x sample buffer (0.125M Tris pH = 6.8, 20% glycerol, 4% SDS, 0.16% bromophenol blue, 10% 2-mercaptoethanol added fresh) and boiled for 10 minutes. 5–10 μl of sample were loaded and run on a 4–15% gradient mini-Protean TGX gel (456–1086, Biorad, Hercules, CA). The gel was then transferred to nitrocellulose membrane and blocked in 3% BSA in TBS plus 0.1% Tween-20 (TBST) for 1 hr at room temperature.

Membranes were then incubated overnight with chicken anti-GFP (1:2000 Aves Labs Cat# GFP-1020, RRID:AB_10000240, Tigard, OR) in blocking solution overnight at 4°C. Following washes with TBST, membranes were incubated with donkey anti-chicken-AP (1:5000, Santa Cruz Biotechnology Cat# sc-2022, RRID:AB_631723, Santa Cruz, CA) in blocking solution for 1 hr at room temperature. After washes, AP was detected with CDP-Star (T2218, Applied Biosystems) and membranes were imaged with a Chemi-Doc system (BioRad).

### Electrophysiology and optogenetic recording

Similar to the previous publication [7], 4-week old mice were anesthetized with a ketamine/xylazine/acepromazine cocktail. Laminectomy was performed, and the spinal cord lumbar segments were removed and placed in ice-cold cutting solution consisting of (in mM) 97 NMDG, 2.5 KCl, 1.25 NaH_2_PO_4_, 30 NaHCO_3_, 20 HEPES, 25 glucose, 2 thiourea, 5 Na-ascorbate, 3 Na-pyruvate, 0.5 CaCl_2_·4H_2_O and 10 MgSO_4_·7H_2_O, titrate pH to 7.3–7.4 with concentrated hydrochloric acid, and an osmolality of 310–320 mOsm, adapted from Ting et al., [8]. Transverse 400 μm lumbar spinal cord slices were prepared using a VT1200S vibratome (Leica Microsystems, Nussloch, Germany) and incubated in 32~34°C cutting solution for 10 min. Slices were incubated in incubation solution consisting of (in mM) 92 NaCl, 2.5 KCl, 1.25 NaH_2_PO_4_, 30 NaHCO_3_, 20 HEPES, 25 glucose, 2 thiourea, 5 Na-ascorbate, 3 Na-pyruvate, 2 CaCl_2_·4H_2_O and 2 MgSO_4_·7H_2_O before recording. The slice was transferred to the recording chamber and continuously perfused with recording solution at a rate of 3–4 ml/min. The recording solution consisted of (in mM) 127 NaCl, 1.8 KCl, 1.2 KH_2_PO_4_, 2.4 CaCl_2_, 1.3 MgSO_4_, 26 NaHCO_3_, and 15 glucose, oxygenated with 95% O_2_ and 5% CO_2_, at a pH of 7.35–7.45 and an osmolality of 300–310 mOsm. Recordings were performed at RT. Spinal cord slices were visualized with an Olympus BX 61WI microscope (Olympus Optical, Tokyo, Japan).

Fluorescently labeled neurons in dorsal horn were identified by epifluorescence and recorded in the whole cell patch-clamp configuration. Glass pipettes (3–5MΩ) were filled with internal solution consisting of (in mM) 120 K-gluconate, 10 KCl, 2 MgATP, 0.5 NaGTP, 20 HEPES, 0.5 EGTA, and 10 phosphocreatine di(tris) salt at a pH of 7.29 and an osmolality of 300mOsm. Blue light (473 nm laser illumination (10 mW, 0.1 ms-1 s, Blue Sky Research, Milpitas, USA)) was delivered through a 40X water-immersion microscope objective to induce light evoked response. All data were acquired using an EPC-9 patch-clamp amplifier and Pulse software (HEKA, Freiburg, Germany). Liquid junction potentials were not corrected. Firing patterns were defined as previously described [9][2]. The series resistance was between 10 and 25MΩ.

## Results

While using Ai32 mice to drive various Cre-dependent expression of ChR2-EYFP, we have generated Cre^+^; *Rosa*^*ChR2-EYFP/ChR2-EYFP*^ (Ai32/Ai32 homozygous) mice to drive high level expression of ChR2-EYFP for optically controlled behavior studies. During these experiments, we noticed off-target expression of ChR2-EYFP. We sought to differentiate whether this was due to ectopic Cre expression or to ‘leaky’ expression from the Ai32 allele. Thus, we examined ChR2-EYFP expression in homozygous Ai32/Ai32 (Cre-negative) mice.

**Fig 1:**
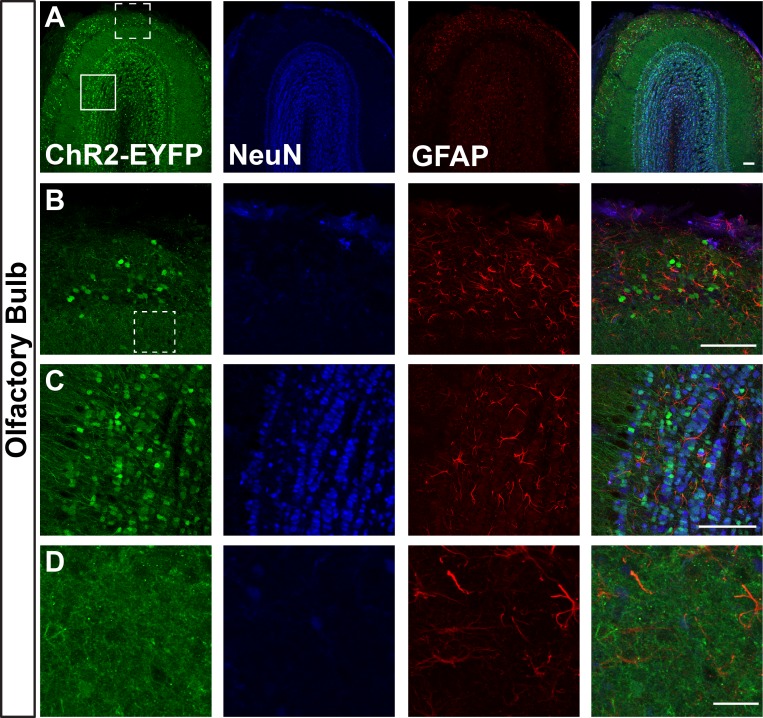
Images of olfactory bulb sections of adult Ai32/Ai32 mice stained with anti-EYFP, anti-NeuN, and anti-GFAP antibodies. B and C show higher magnification images of dotted line (B) and solid line (C) boxes in A. B shows the glomerular cell layer, C shows mitral/granule cell layer. D shows a ‘negative’ region not showing leaky ChR2 expression (dotted line boxed region in B). n = 3 mice, scale bars = A-C 100 μm, D 25 μm.

**Fig 2:**
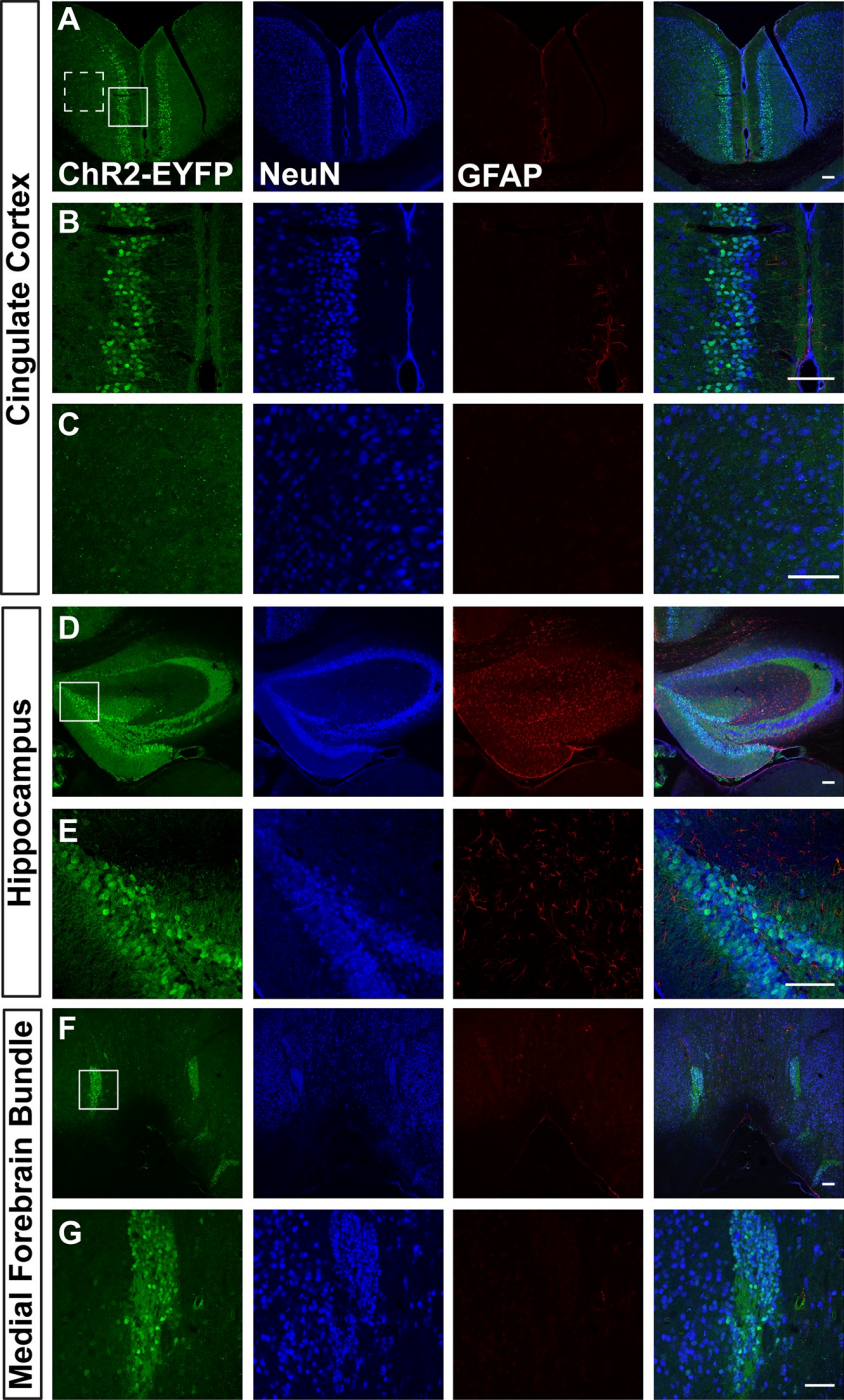
Images of cingulate cortex (A-C), hippocampus (D&E), and the medial forebrain bundle and islands of Calleja in the olfactory tubercle (F&G) sections in adult Ai32/Ai32 mice stained with anti-GFP, anti-NeuN, and anti-GFAP antibodies. B and C are higher magnification ChR2 expressing (B) and non-expressing (C) regions from A (solid line and dotted line boxes, respectively). E and G are higher magnification images of regions boxed in D and F respectively. n = 3 mice, scale bars = 100 μm.

**Fig 3:**
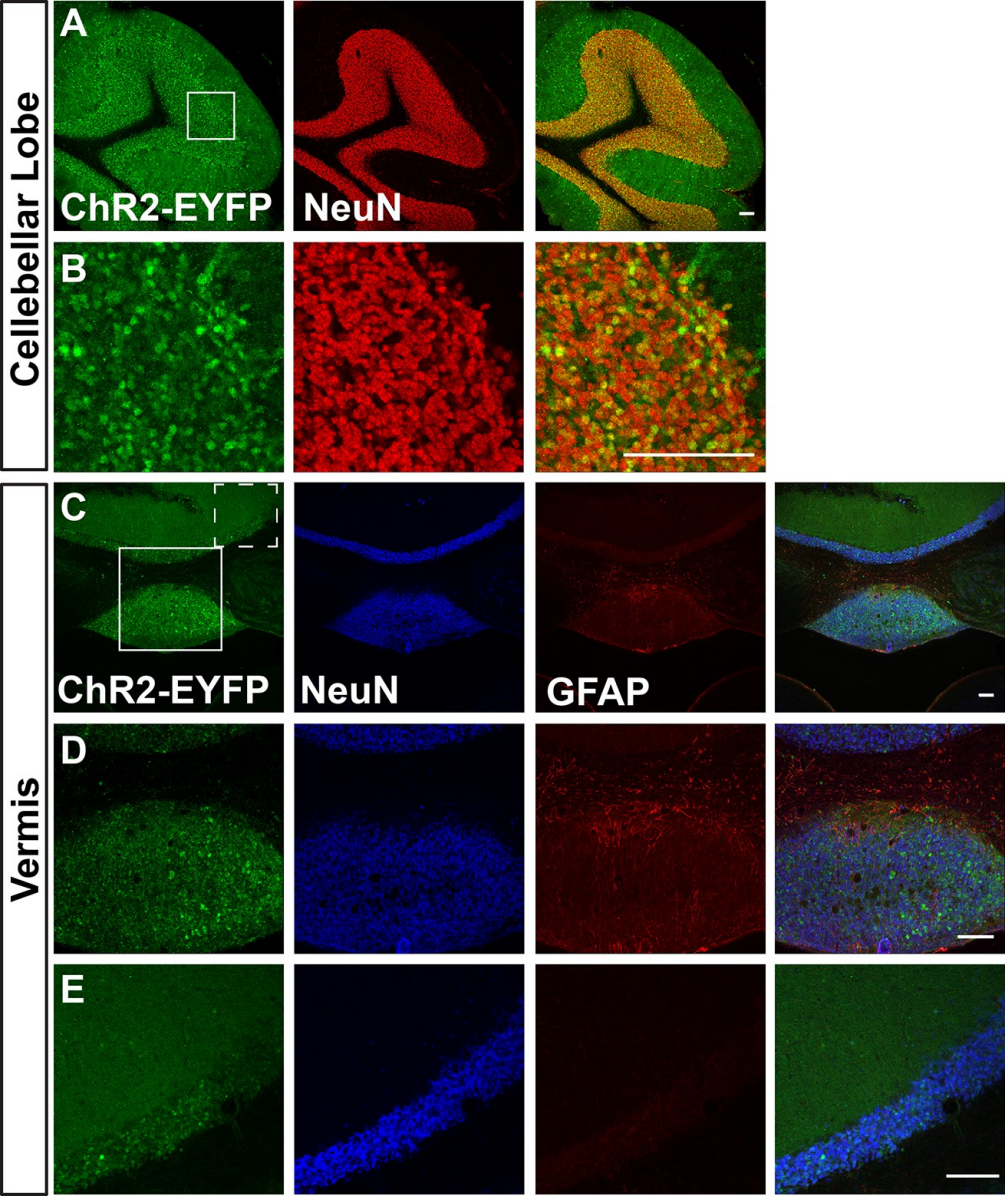
Images of cerebellar lobe (A&B) and vermis (C-E) sections of adult Ai32/Ai32 mice immunostained with anti-GFP, anti-NeuN and anti-GFAP antibodies. B shows a higher magnification view of region boxed in A. D and E show ChR2 expressing (D) and non-expressing (E) regions boxed in C (solid line and dotted line boxes, respectively). n = 3 mice, scale bars = 100 μm.

**Fig 4:**
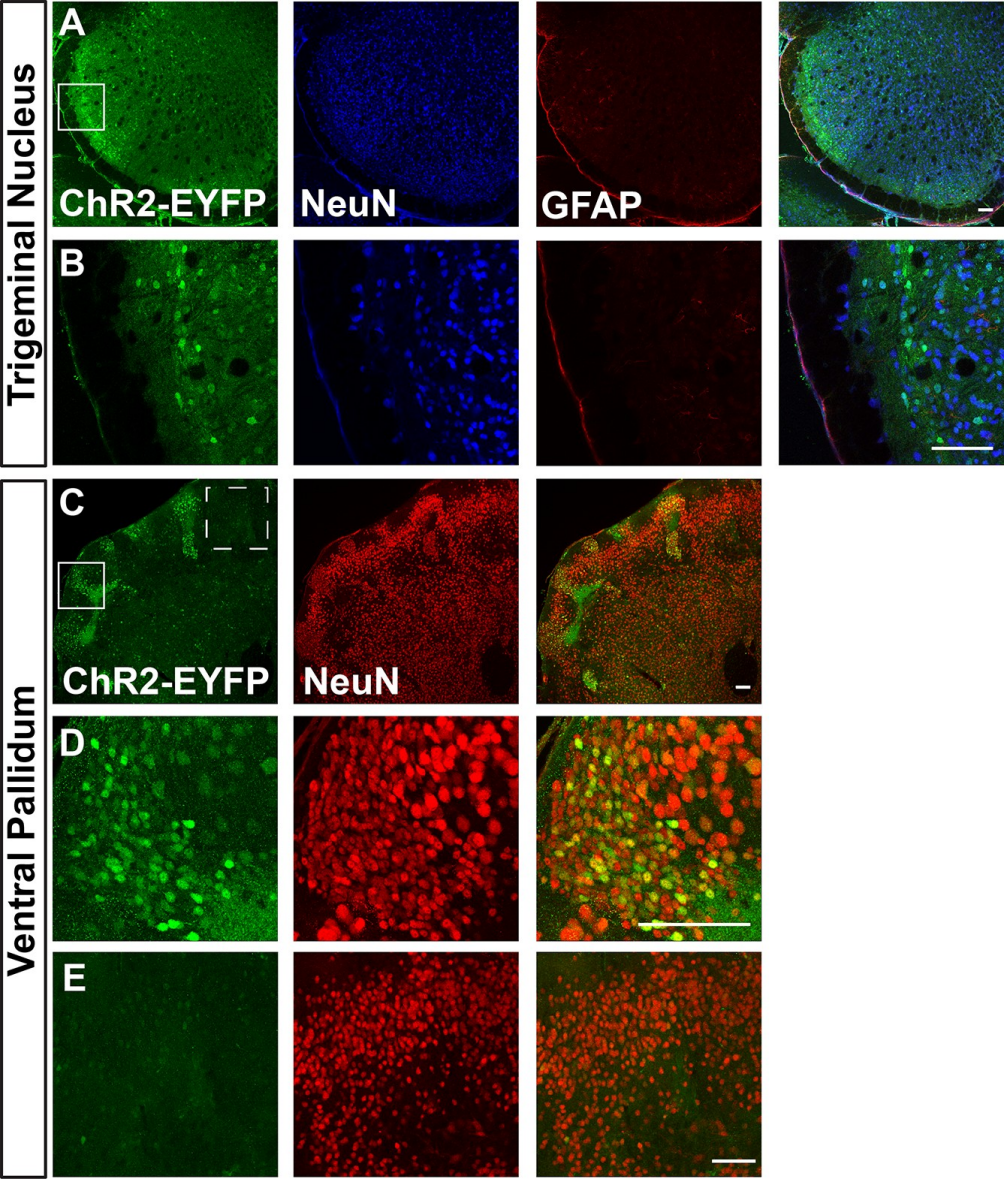
Images of brainstem spinal trigeminal nucleus caudalis (A&B) and ventral pallidum (C-E) sections in adult Ai32/Ai32 mice stained with anti-GFP and anti-NeuN antibodies. B shows a higher magnification view of region boxed in A. D and E show ChR2 expressing (D) and non-expressing (E) regions boxed in C (solid line and dotted line boxes, respectively). n = 3 mice, scale bars = 100 μm.

We first visualized ChR2-EYFP expression in Ai32/Ai32 mice using immunostaining against EYFP. We found leaky EYFP signals throughout the nervous system of Ai32 homozygous mice, and this expression was particularly high in a number of areas. Fig 1 shows representative immunostaining of Ai32/Ai32 adult olfactory bulb (OB). We found leaky expression of EFYP in the granular, mitral, and glomerular cell layers (Fig 1A–1C). EFYP+ cells express the neuronal marker NeuN in the mitral and granular layers (Fig 1C) but do not express the astrocyte marker GFAP in the OB (Fig 1B & 1C). Figs 2–4 show leaky expression of EYFP in NeuN+ cells of the cingulate cortex (Fig 2A & 2B), hippocampus (Fig 2C & 2D), the islands of the Calleja in the olfactory tubercle (Fig 2E & 2F), in the cerebellar lobes (Fig 3A & 3B) and in the vermis (Fig 3C & 3D). In the brainstem, we saw EYFP expression in NeuN+ cells of the spinal trigeminal nucleus caudalis (Fig 4A & 4B), and in isolated regions of the ventral pallidum (Fig 4C & 4D).

**Fig 5:**
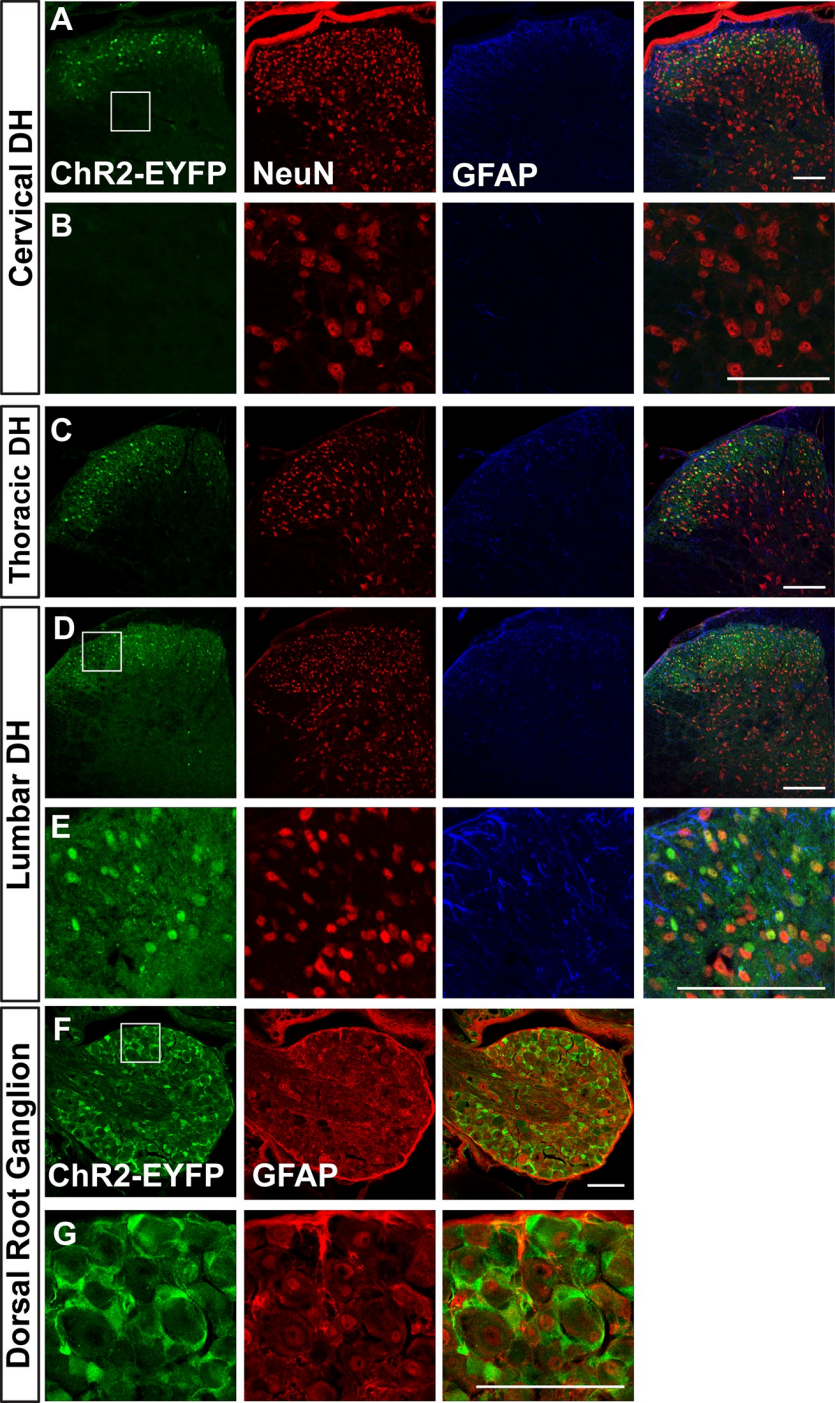
A. Images of cervical dorsal horn (DH) (A&B), thoracic DH (C), lumbar DH (D&E) and dorsal root ganglion (F&G) of adult Ai32/Ai32 mice stained with anti-GFP, anti-NeuN, and anti-GFAP antibodies. B shows a higher magnification view of ChR2 non-expressing region boxed in A. E and G show higher magnification views of regions boxed in D, F respectively. n = 3 mice, scale bars = 100 μm.

We further characterized leaky expression of ChR2-EFYP in the dorsal root ganglion (DRG) and spinal cord dorsal horn (Fig 5). Leaky EYFP signals are found in NeuN+ interneurons of the cervical, thoracic, and lumbar dorsal horn of Ai32/Ai32 mice (Fig 5A–5E). Interestingly, in DRGs, EYFP is expressed in the GFAP+ satellite glia cells, but not in primary afferent neurons themselves (Fig 5F & 5G). In summary, this leaky expression occurs in both neurons and glial cells in many regions of the nervous system in homozygous Ai32/Ai32 (Cre-negative) mice.

**Fig 6:**
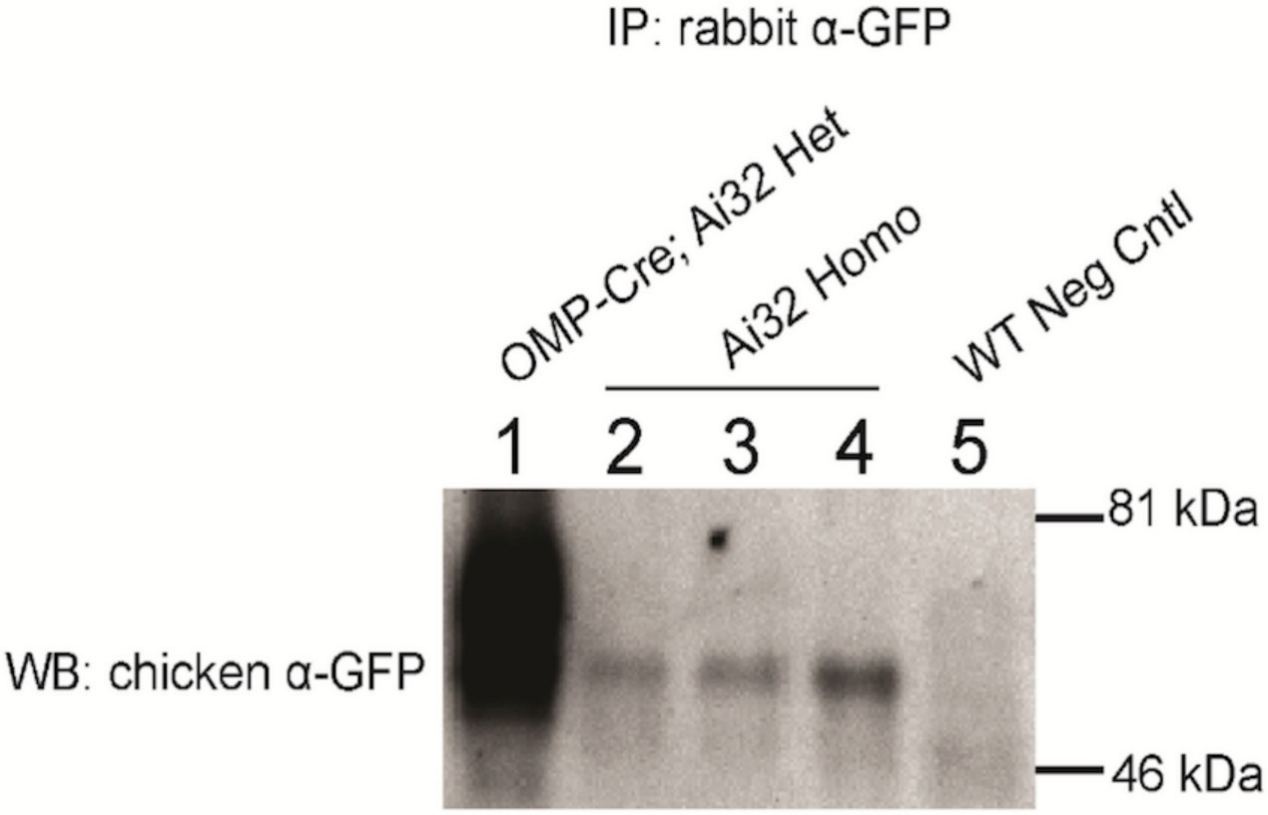
Western blot analysis of ChR2 (H134R)-EYFP expression in *Ai32* mice. Olfactory bulb (OB) cell lysates were immunoprecipitated using a rabbit anti-GFP antibody and blotted with a chicken anti-GFP antibody. Lane 1: Positive control *Omp*^*Cre*^; *Ai32*, lane 2,3,4: *Rosa*^*ChR2-EYFP/ChR2-EYFP*^ homozygous *Ai32* mice (n = 3 mice.), lane 5: Negative control CDI mouse. ChR2-EYFP = ~62 kDa.

Following immunostaining, we performed western blotting to confirm that EYFP fluorescence corresponds to the expression of full-length ChR2-EYFP protein. Fig 6 shows western blot detection of ChR2-EYFP expression in Ai32/Ai32 OB compared to the positive (*Omp*^*Cre*^*;Ai32/Ai32*) and negative control mice. OB cell lysates were immunoprecipitated using rabbit anti-GFP antibody and blotted using chicken anti-GFP (OB lysates were chosen because of the high degree of fluorescence in immunostaining, Fig 1). Ai32/Ai32 OB lysates showed a ~60kDa positive band, which is as anticipated (ChR2-EYFP predicted weight = 62 kDa) and similar in size to the positive control ChR2-EYFP from *Omp*^*Cre*^; Ai32 OB lysates (Fig 6 lanes 1–4). This band is absent from negative control CD1 (Ai32 negative) OB lysate (Fig 6 lane 5). This result confirms that the fluorescence visualized as leaky expression corresponded to full length ChR2-EYFP protein.

**Fig 7:**
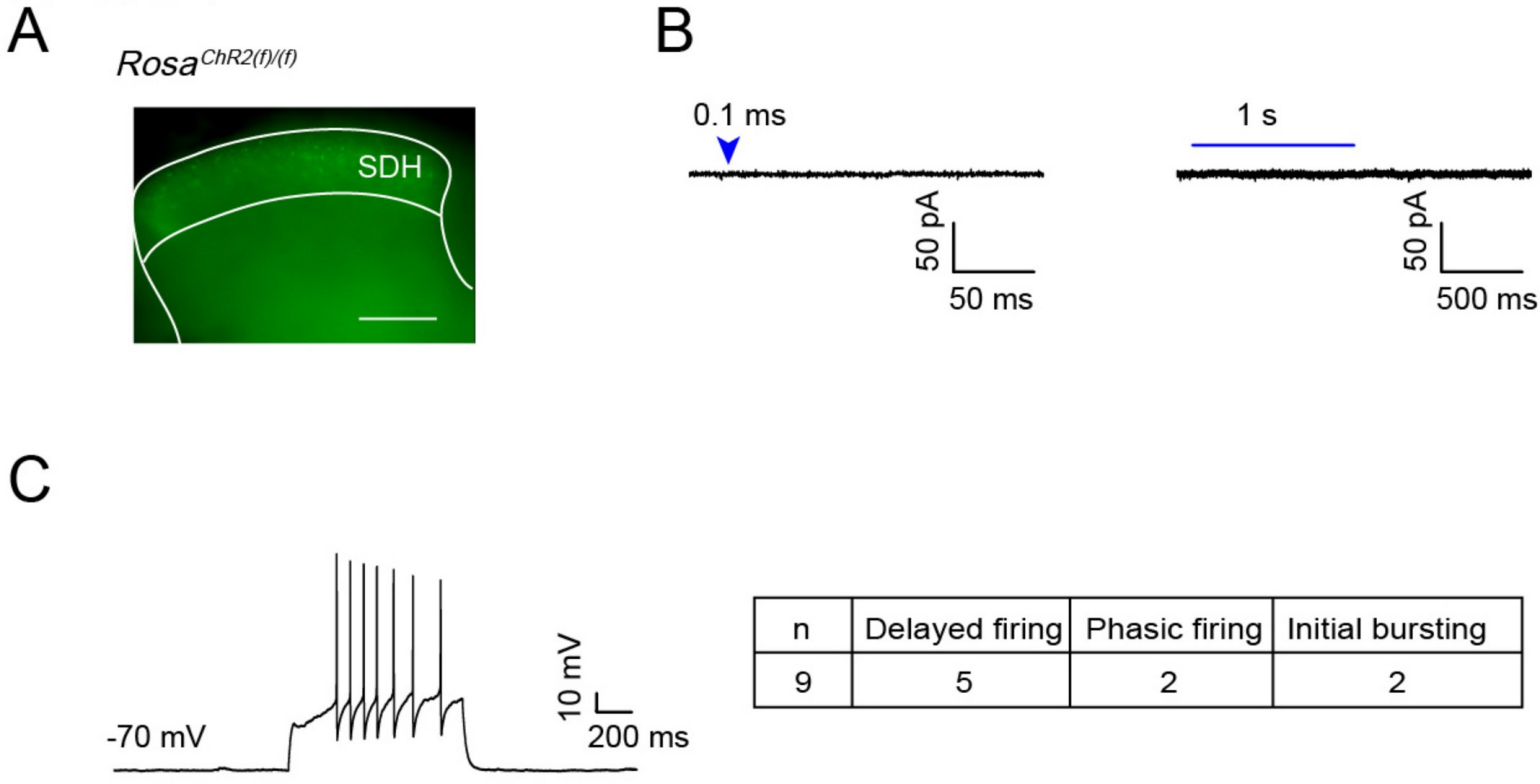
Leaky expression of ChR2(H134R)-EYFP in dorsal spinal cord neurons of four-week-old Ai32 mice is not sufficient to generate photocurrents. (A) ChR2-EYFP expression in dorsal horn of lumbar spinal cord slice from 4 pw Ai32/Ai32 mice (scale bar 0.1 mm). (B) Whole cell recording from 12 of ChR2-EYFP expressing superficial dorsal horn (SDH) cells, light stimulation (0.1 ms or 1 s) did not induce any currents. (C) Representative trace of delayed firing in ChR2-EYFP expressing cell by 50 pA current injection. Table shows firing patterns of recorded cells: 5 cells showed delayed firing pattern (DFN), 2 cells showed phasic firing pattern (PFN), 2 cells showed initial bursting firing pattern (IBN).

To test whether the leaky expression of ChR2-EYFP is functional, we examined light induced responses in ChR2-EYFP expressing dorsal horn cells in the spinal cord slices from 4-week old Ai32/Ai32 mice (Fig 7A). Out of 12 recorded cells, none showed detectable response upon light stimulation (0.1 ms—1 s duration). Based on our previous experience [3], 0.1ms blue laser pulse durations are sufficient to induce photocurrents in Cre-driven ChR2 expressing spinal cord DH neurons. In addition, these recorded cells are viable and responsive, as they show action potential firing upon current injection (Fig 7C). Thus, this result indicates that leaky ChR2-EYFP expression in dorsal spinal cord neurons is not high enough to generate detectable photocurrents (Fig 7B) at four weeks of age. However, our results do not exclude the possibility that this leaky expression of ChR2 could generate photocurrents with stronger light stimulation or with potentially higher expression levels in adult mice.

## Discussion

We report Cre-independent, leaky ChR2-EYFP expression in Ai32/Ai32 homozygous mice as identified by EYFP immunostaining and IP/western blotting. While this expression occurred throughout the nervous system, we identified regions with particularly high ChR2-EYFP expression including the olfactory bulb, hippocampus, cerebellum, and other regions. While anti-EFYP immunostaining allows for clear identification of this off-target expression, the level of this expression is likely to be much lower compared to Cre-driven Ai32 ChR2-EYFP. Indeed, our western blotting results reveal much lower amounts of ChR2-EYFP purified from Ai32/Ai32 (Cre-negative) OB lysates in comparison with *Omp*^*Cre*^-driven positive control tissue (Fig 6). While previous work has reported no Cre-independent ChR2-EFYP mRNA expression in this line [4], it is possible that the off-target leaky expression we found is related to the use of Ai32/Ai32 homozygous tissue. We also found that, although the leaky expression of ChR2-EFYP is obvious in some areas of nervous system, this expression is not sufficient to generate a detectable photo current at least in the spinal cord of 4-week old mice, likely due to the low level of ChR2 present in these cells.

The Ai32 line uses a CAG promoter cassette organization designed to drive high levels of transgene expression. The off-target expression we found is possibly a consequence of ‘read-through’ transmission of the floxed stop element, especially given the strong promoter used to drive this cassette. Notably, other lines using this cassette organization have been reported to show off-target transgene mRNA expression [4]. While read-through transcription of similar floxed stop elements has been reported in other Cre-reporter transgene systems [10,11], such transcription has been shown to be considerably weaker than expression after Cre-mediated stop element excision, consistent with our results. It should be noted that, during propagation, the Ai32 line used in this study have been crossed to other lines, and this genetic background variability may contribute to this off-target leaky expression of ChR2-EFYP. The presence of this leaky ChR2-EYFP expression is an important consideration for labs using this line, specifically in the use of EYFP immunostaining to characterize ChR2 expression in *Ai32* mice. Further, while we did not see functional photocurrents in our recordings with spinal cord dorsal horn neurons at 4 week old, we cannot rule out the possibility of functional effects of leaky ChR2 expression after light stimulation either through some modulatory effect or by direct activation in cells with higher expression levels in adult mice.
